# Exploring pharmacists’ attitude, willingness and barriers to provide extended community pharmacy services: Implications for improved pharmacy services

**DOI:** 10.1371/journal.pone.0310141

**Published:** 2024-09-09

**Authors:** Anan S. Jarab, Walid Al-Qerem, Karem H. Alzoubi, Nadeen Almomani, Shrouq Abu Heshmeh, Tareq Mukattash, Yazid N. Alhamarneh

**Affiliations:** 1 College of Pharmacy, Al Ain University, Abu Dhabi, United Arab Emirates; 2 Faculty of Pharmacy, Department of Clinical Pharmacy, Jordan University of Science and Technology, Irbid, Jordan; 3 Faculty of Pharmacy, Department of Pharmacy, Al-Zaytoonah University of Jordan, Amman, Jordan; 4 Department of Pharmacy Practice and Pharmacotherapeutics, University of Sharjah, Sharjah, United Arab Emirates; 5 Faculty of Pharmacy, Jordan University of Science and Technology, Irbid, Jordan; 6 Faculty of Medicine and Dentistry, Department of Pharmacology, University of Alberta, Edmonton, Cananda; UCSI University, MALAYSIA

## Abstract

**Objective:**

This study aimed to evaluate pharmacists’ attitude and willingness to provide extended community pharmacy services (ECPS), the barriers to ECPS, and the factors associated with attitude and willingness to implement ECPS.

**Methods:**

In this cross-sectional study, a validated, self-administered questionnaire was distributed online to community pharmacists across the United Arab Emirates between September and November 2023. In addition to sociodemographic data, the questionnaire evaluated attitudes toward ECPS, willingness to provide ECPS, and barriers to its implementation. Binary logistic regression was conducted to explore the factors associated with attitude and willingness to implement ECPS.

**Results:**

The study included 409 pharmacists. Over half of the participants reported below the median attitude (58.7%) and willingness (59.4%) scores. Female pharmacists had a lower attitude towards ECPS (OR = 0.425, 95%CI: 0.242–0.747). Higher number of daily prescriptions (OR = 1.066, 95%CI: 1.029–1.105) and being a Pharm D graduate (OR = 2.664, 95%CI: 1.439–4.932) were associated with higher willingness to provide ECPS, while an increased number of employed pharmacists (OR = 0.518, 95%CI: 0.397–0.676) was associated with a lower willingness (OR = 0.049, 95%CI: 0.004–0.660). Patients’ preoccupation (76.5%), lack of specific training (76.3%), lack of clinical problem-solving skills (74.6%) and lack of a private consultation room (74.6%) were the most commonly reported barriers to ECPS.

**Conclusions:**

Community pharmacists demonstrated unsatisfactory levels of attitude and willingness towards ECPS provision. Therefore, providing training and education programs that enhance pharmacists’ perception and willingness to implement ECPS and emphasize improving clinical problem-solving skills, as well as setting up specific private consultation rooms, is deemed necessary. Female pharmacy graduates, pharmacists dispensing fewer daily prescriptions, and those working with a higher number of employed pharmacists should be specifically targeted when implementing these strategies.

## Introduction

The scope of the pharmacy profession has recently evolved as a result of pharmacists working in close contact with patients [1]. Previous evidence supported the invaluable role of community pharmacists in providing patient care in a variety of settings, including identifying issues related to drug therapy, patient counseling, and improving patients’ quality of life [2–5].

Extended community pharmacy services (ECPS) refer to additional healthcare services that pharmacists can offer to their patients beyond the traditional dispensing of medications [6,7]. ECPS are becoming increasingly important as pharmacists play a larger role in providing various services related to primary patient care, such as medication therapy management, patient counseling, chronic disease management, smoking cessation programs, and point-of-care testing such as blood glucose or blood cholesterol testing [8]. A previous study found that the general population in Ethiopia had a high acceptance rate of various ECPS, such as blood pressure, blood glucose, and lipid level measurements. The participants reported several reasons for their positive response, including the lack of need for appointments, shorter waiting times compared to primary care centers, and the convenience of proximity to their residences [6]. These services can help improve patient outcomes, reduce healthcare costs, and enhance the overall quality of care [9]. In addition to patients, pharmacists can also profit from ECPS in a variety of ways, such as by improving their professional standing and sense of fulfillment in their line of work. However, it might result in a heavier burden for pharmacists and a potentially tense working relationship with physicians [10]. A systematic review found that pharmaceutical care services and healthcare promotion services were the most frequently provided ECPS, with both pharmacists and the public holding positive perceptions towards them. However, challenges such as a lack of time and staff shortages hinder the implementation of these services [11].

In developed countries like the USA, community pharmacists showed wide practice of ECPS, where they have the permission to administer vaccines, prescribe medications for some chronic diseases, perform laboratory testing, and create care plans [12]. In comparison, ECPS in developing countries is not well-practiced [10]. ECPS are either infrequently employed or not employed at all in the United Arab Emirates (UAE) and a number of other Arab nations that share almost the same community pharmacy practice scenario [10]. According to a study conducted in Saudi Arabia, most of the pharmacists have never provided ECPS, including health screening services, vaccination services, and medication therapy management services [13]. Researchers in the UAE discovered that the influence of pharmacists on pharmaceutical practice was limited, with only 9.2% of them showing a positive response to reducing prescribing errors and identifying drug-related problems [14]. In spite of the general public’s positive perspectives and strong beliefs on the importance of ECPS provision [15], several variables were found to affect the implementation of these services, such as pharmacists’ age, years of experience, lack of interest or urgency on the part of the patients, time constraints, unclear understanding of the pharmacists’ professional role, communication barriers, and lack of financial benefits[14]. Therefore, it is crucial to investigate pharmacists’ perceptions and willingness to provide such services in the presence of many obstacles to their implementation, because positive views and high readiness towards ECPS will enhance the provision of these services.

In addition to the paucity of research findings, the present research is the first to evaluate pharmacists’ attitudes and willingness to provide ECPS, to assess the barriers to ECPS implementation, and to explore the factors associated with a lower attitude and willingness to implement such services in the UAE. The findings of this study should aid in the development of coordinated approaches from all stakeholders, including the government, healthcare providers, and patients, to surmount the obstacles that stand in the way of ECPS implementation.

## Materials and methods

### Study design and subjects

In this cross-sectional study, a validated questionnaire was distributed as a Google Form via social media sites such as Facebook, WhatsApp, and Twitter to pharmacists working in different community pharmacy settings across the UAE using convenience sampling in the period from September through November 2023. Pharmacists were included in the study if they were graduates of universities recognized by the Ministry of Higher Education and authorized to practice as pharmacists in the UAE.

### Study instrument

The current self-administered, online-based survey was developed after a review of the relevant literature [16,17]. The survey started with an introduction outlining the aims of the study and emphasizing the anonymity of the participants. The first part of the questionnaire collected socio-demographic and work environment-related information. The second 11-item part evaluated pharmacists’ attitude towards ECPS on a 5-point Likert scale ranging from strongly disagree (1 point) to strongly agree (5 point), with a maximum possible score of 55. The third 6-item part evaluated willingness to implement ESCAP using yes/no questions, and each positive response received one point. In the last section, 22 elements evaluated the obstacles that pharmacists face in providing ECPS. Scores below the median score of attitude and willingness were labelled as “low”, while scores above the median were labelled as “high”. An expert panel including two academic professors of pharmacy practice and two community pharmacists evaluated the content validity of the questionnaire to ensure its relevancy and comprehensiveness. Next, the survey was piloted with ten community pharmacists to assess its clarity, appropriateness, and feasibility. The data obtained from the pilot test was not ‎included in the final data analysis.

### Ethics approval

The current research received the required ethical approval from the research ethics committee at Al Ain University- Abu-Dhabi Campus (Ref. No. COP/AREC/AD/32). Pharmacists who agreed to participate were requested to select the option “I have read the study information and I agree to participate,” which represented an informed consent to participate, before moving on to the survey questionnaire.

### Statistical analysis

Statistical analysis was conducted using SPSS version 28 from IBM (Illinois, New York, USA) [18]. Categorical variables were presented as frequencies and percentages. The Kolmogorov- Smirnov test revealed that the data were not normally distributed, therefore, continuous variables were presented as median and 95% Cl, and the dependent variables, including attitude and willingness scores, were dichotomized based on the median score of each outcome. In the bivariate analyses, the Chi-squared test was used to assess the association between different categorical variables and the dichotomized attitude and willingness scores, while the Mann-Whitney U test was used to investigate the association between different not-normally distributed continuous variables and the dichotomized attitude and willingness scores. Variables that had a p-value <0.2 in the bivariate analyses were included as independent variables in the binary logistic regression analysis. Significance was determined at a p-value < 0.05.

## Results

The present study enrolled 409 pharmacists with a median age of 27 (27–28) years. The majority of the pharmacists were female (78.2%), had a pharmacy degree (80.9%), were working in independent pharmacies (80.5%) and had an internet connection (95.8%). The median for work experience was 3 years (3–4), and the average number of visiting patients was 35 (35–40). Table 1 describes the sociodemographic characteristics of the participating pharmacists.

**Table 1:** Sociodemographic characteristics of the participating pharmacists (n = 409).

Characteristics		Median (95%Cl) or Frequency (%)
Age		27 (27–28)
Gender	Male	89 (21.8)
	Female	320 (78.2)
Work experience (years)		3 (3–4)
weekly working hours		48 (48–56)
Average number of patients served a day		35 (35–40)
Average number of prescriptions dispensed per day		15 (15–20)
Number of pharmacists working in the pharmacy		2 (2–3)
Average time spent with each patient (min)		5 (5–6)
Education background	Pharmacy	331 (80.9)
	Pharm D	78 (19.1)
Access to the internet	No	17 (4.2)
	Yes	392 (95.8)
Pharmacy type	Chain community pharmacy	79 (19.5)
	Independent community pharmacy	326 (80.5)

The median of the attitude’s score was 36 (36–37) out of the maximum possible score of 55. Hence, 240 (58.7%) of the enrolled participants were in the low attitude group, and 169 (41.3%) were in the high attitude group. More than three-quarters of the participants strongly agreed or agreed that ECPS might generate extra revenue (81.1%) and require major upskilling of clinical knowledge (77.7%). On the other hand, a very small number of pharmacists strongly disagreed or disagreed that doctors and other health professionals will not support an ECPS role for pharmacists (7.8%), and that pharmacy education never provided them with the skills needed for ECPS (15.6%). Participants’ responses to the attitudes toward implementing ECPS’s are presented in Table 2.

**Table 2:** Pharmacists’ attitude toward implementing ECPS (n = 409).

Item	Frequency (%)				
	Strongly disagree	Disagree	Neutral	Agree	Strongly agree
Every CP must provide ECPS	14 (3.4)	34 (8.3)	135 (33)	134 (32.8)	92 (22.5)
I fully support the concept of ECPS	13 (3.2)	34 (8.3)	123 (30.1)	138 (33.7)	101 (24.7)
ECPS is really the doctor’s role ^a^	10 (2.4)	114 (27.9)	92 (22.5)	152 (37.2)	41 (10)
ECPS requires major up skilling of clinical knowledge	4 (1)	11 (2.7)	76 (18.6)	239 (58.4)	79 (19.3)
Doctors and other health professionals will not support an ECPS role for pharmacists ^a^	5 (1.2)	27 (6.6)	130 (31.8)	186 (45.5)	61 (14.9)
CPs alone cannot provide ECPS ^a^	4 (1)	99 (24.2)	91 (22.2)	199 (48.7)	16 (3.9)
My pharmacy education never provided me with skills needed for ECPS ^a^	5 (1.2)	71 (14.4)	200 (48.9)	127 (31.1)	6 (1.5)
I may lose my patients if I start providing ECPS to them ^a^	18 (14.4)	209 (51.1)	97 (23.7)	76 (18.6)	9 (2.2)
ECPS is good to market pharmacy services	2 (0.5)	15 (3.7)	53 (13)	228 (55.7)	57 (13.9)
ECPS can be used to generate extra revenue	0 (0)	25 (6.1)	52 (12.7)	246 (64.5)	68 (16.6)
ECPS is difficult to implement due to language barrier ^a^	20 (4.9)	72 (17.6)	171 (41.8)	132 (32.3)	14 (3.4)

^a^ Reverse coding (negative statement).

The median of the willingness score was five (5–6) out of the maximum possible score of six. Thus, 243 (59.4%) of the participants were in the low willingness group, and 166 (40.6%) were in the high willingness group. The participants demonstrated the highest willingness to measure blood pressure (96.6%), followed by blood sugar testing (94.1%), ear piercing (86.6%), calculating body mass index (71.4%), and pregnancy testing (69.7%), while they were least willing to do body fat analysis (52.6%).

Results of the bivariate analysis using the Chi-squared and Mann-Whitney U-test revealed that variables including sex, average number of daily-dispensed prescriptions, number of pharmacists, and the education background had a P value < 0.2 and therefore were included in the bivariate logistic regression analysis.

As shown in Table 3, the results of the binary logistic regression analysis showed that females had fewer odds of having a positive attitude towards ECPS (OR = 0.425, 95%CI:0.242–0.747). A higher number of daily prescriptions (OR = 1.066, 95%CI: 1.029–1.105) and being a Pharm D graduate (OR = 2.664, 95%CI: 1.439–4.932) increased the odds of having a high willingness to provide ECPS. On the other hand, an increased number of employed pharmacists decreased the odds of willingness to provide ECPS (OR = 0.518, 95%CI: 0.397–0.676).

**Table 3:** Factors associated with attitude towards ECPS and the willingness for its implementation.

Variable			Attitude				Willingness			
			P	OR	95% C.I for OR				95% C.I for OR	
					Lower	Upper	P	OR	Lower	Upper
Sex	Female		0.003	0.425	0.242	0.747	0.198	0.660	0.351	1.242
	Male (REF)		0	.	.	.	0	.	.	.
Average number of prescriptions dispensed per day			0.349	1.015	0.984	1.046	<0.001	1.066	1.029	1.105
Number of pharmacists in the community pharmacy			0.277	0.928	0.811	1.062	<0.001	0.518	0.397	0.676
Education background		Pharm D	0.574	0.855	0.494	1.478	0.002	2.664	1.439	4.932
		Pharmacy (REF)	0	.	.	.	0	.	.	.

As shown in Table 4, the most recognized barriers to the implementation of ECPS were that patients are usually busy (76.5%), a lack of specific training (76.3%), a lack of clinical problem-solving skills (74.6%), and a lack of a private consultation room (74.6%).

**Table 4:** Barriers to implement ECPS (n = 409).

Item	Frequency (%)		
	Disagree	Not sure	Agree
Other pharmacists’ attitudes towards ECPS	22 (5.3)	156 (38.1)	231 (56.4)
Fear of change	35 (8.6)	86 (21)	288 (70.4)
Lack of motivation	26 (6.3)	95 (23.3)	287 (70.2)
Lack of confidence	42 (10.3)	81 (19.8)	286 (69.9)
Lack of incentive for employee pharmacists	13 (3.2)	141 (34.5)	255 (62.3)
Lack of clinical problem-solving skills	22 (5.3)	82 (20)	305 (74.6)
Lack of communication skills	127 (31.1)	73 (17.8)	184 (45)
Lack of specific training	16 (3.9)	81 (19.8)	312 (76.3)
Lack of documentation (processes/software)	27 (6.6)	138 (33.7)	244 (59.7)
Lack of drug information resources (processes/access)	60 (14.7)	160 (39.1)	189 (46.2)
Insufficient finances	25 (6.1)	108 (26.4)	276 (67.5)
Inappropriate physical space	22 (5.3)	85 (20.8)	302 (73.8)
Lack of personal motivation	76 (18.6)	98 (24)	235 (57.5)
Inappropriate management system and poor work flow	8 (2)	117 (28.6)	284 (69.4)
Lack of patient demand	96 (23.5)	90 (22)	223 (54.5)
Doctor/nurse resistance	35 (8.6)	128 (31.3)	246 (60.1)
Lack of access to patient medical records	19 (4.6)	126 (30.8)	264 (64.5)
Lack of data on value of ECPS	39 (9.5)	123 (30.1)	247 (60.4)
Patients are not willing	59 (14.4)	104 (25.4)	246 (60.1)
Patients are usually busy	9 (2.2)	87 (21.3)	313 (76.5)
Non-willingness for payment	17 (4.2)	111(27.1)	281 (68.7)
Lack of private consultation room	24 (5.9)	78 (19.2)	305 (74.6)

## Discussion

Numerous professional organizations for pharmacists are working to improve the quality of patient-centered care provided by community pharmacies globally [19,20]. One of the best approaches for the advancement of community pharmacy practice is the establishment of ECPS [21]. However, it is imperative to examine how pharmacists perceive and are willing to offer these services, as well as the obstacles and variables preventing their implementation, which was the main purpose of this study.

Most of the current study participants showed unsatisfactory levels of attitudes and willingness towards ECPS implementation, with several identified barriers. Female gender was significantly associated with a negative attitude, whereas a lower number of daily prescriptions, having a pharmacy degree, and a higher number of employed pharmacists significantly reduced the willingness to provide ECPS.

When compared with the present study, earlier research findings reported a better attitude among community pharmacists in this regard [16,17,22,23]. Although the majority of the current study participants perceived that ECPS could be used to generate extra revenue, they believed that healthcare providers would not support the ECPS role of pharmacists. In comparison, more pharmacists who were enrolled in an earlier study disagreed with this statement [17]. Furthermore, most of the participants in the current study thought that ECPS requires major upskilling of clinical knowledge (77.7%), and that pharmacy education never provided them with the skills needed for ECPS (84.4%). In comparison, 87.7% of the pharmacists who took part in an earlier study acknowledged that ECPS needs upskilling in clinical knowledge. Nonetheless, only 25.6% thought they had never acquired the skills necessary for ECPS during their pharmacy education [17]. These findings clearly demonstrate the need for targeted interventions to enhance pharmacists’ attitudes toward expanding their services, especially when considering the assistance of other healthcare providers and the requirement for clinical knowledge upskilling. It is deemed necessary to support pharmacists’ collaboration with other healthcare professionals in order to establish a welcoming atmosphere for ECPS. It is also crucial to design and implement continuing education initiatives that give pharmacists advanced clinical knowledge related to ECPS, thus bridging the knowledge gap that exists between present education and the skills for expanded roles.

The present study found that females had a significantly lower attitude toward ECPS than males. Female pharmacists tend to perform more domestic caregiving duties and have less time to dedicate to ECPS. Hence, their attitudes toward ECPS may be influenced by the notion that this service will necessitate more time and effort, potentially affecting their work-life balance.

The majority of the current study participants expressed a low willingness to implement ECPS. On the other hand, several other studies reported a high willingness to provide ECPS among community pharmacists [17,24]. Although more than 90% of the participants in our study showed a high willingness to perform blood pressure measurement and blood glucose testing, only 52.6% were willing to conduct body fat analysis. Similar results were found in an earlier study [17]. Previous literature reported that most communities support the introduction of the extended roles of community pharmacists and the provision of ECPS [6,25–27].

Results revealed that pharmacists who handled more prescriptions and those who worked with fewer pharmacy staff showed a noticeably higher willingness to provide ECPS. Given their heavy workload and the wide range of cases they encounter daily, it is feasible that such pharmacists developed awareness of patients’ interests and what benefits their health. Thus, regardless of the number of prescriptions, pharmacists might be more willing to provide ECPS because they may be more conscious of its benefits and more confident in their ability to provide it. Additionally, a smaller workforce at the pharmacy might have led to pharmacists taking on more duties and developing a stronger sense of obligation to serve the community, which would have increased their willingness to implement ECPS.

Pharm D graduates demonstrated a higher willingness to provide ECPS than B Pharm holders in this study. Pharm D graduates typically receive more extensive training in clinical pharmacy practice, patient counseling, and pharmacotherapy management than pharmacists with a Bachelor of Pharmacy degree [28]. Thus, the increased willingness to provide extended services may be due to the expanded scope of practice that is inherent in the Pharm D curriculum because Pharm D programs place a greater emphasis on patient care and inter-professional collaboration, which may encourage graduates to seek out opportunities to provide a higher level of care to patients.

In the current study, the most commonly recognized barriers to the implementation of ECPS were patients’ preoccupation, a lack of specific training, a lack of clinical problem-solving skills, and a lack of a private consultation room. A previous study reported that lack of time by customers to interact with the pharmacist was the barrier to providing health-promotion and preventive services in community pharmacies [29]. A systematic review reported that lack of knowledge, skills, and time were the most common barriers to performing ECPS [8]. In Pakistan, even though the majority of the participants were unaware of pharmaceutical care, they were willing to accept a change in practice if given the necessary knowledge and training [16]. Pharmacists’ concerns about the availability of pharmacy consultation rooms were reported as a barrier to the provision of ECPS in another review [30]. Lack of a counseling space was also identified as a barrier in other studies conducted in Canada [29], South Africa [31], Malaysia [32,33], and Jordan [34].

The current study has some limitations. The cross-sectional study design cannot confirm the cause-and-effect relationship. Furthermore, the use of the self-report method used in the study survey may expose the results to social-desirability bias.

## Conclusions

Pharmacists’ attitudes and willingness to provide ECPS need improvement, and there are several barriers to overcome. Hence, it is advisable to implement training and educational programs that improve clinical problem-solving skills and fill the gap in unsatisfactory pharmacy education. Establishing private consultation rooms in pharmacies is also advised in order to support private patient-pharmacist interactions, which are crucial for the efficient administration of ECPS. When putting these strategies into practice, special attention should be paid to female pharmacists, those who have fewer daily prescriptions, bachelor’s pharmacy holders, and pharmacists working in pharmacies with a higher number of employed pharmacists.
